# The role of RNA folding free energy in the evolution of the polymerase genes of the influenza A virus

**DOI:** 10.1186/gb-2009-10-2-r18

**Published:** 2009-02-12

**Authors:** Rachel Brower-Sinning, Donald M Carter, Corey J Crevar, Elodie Ghedin, Ted M Ross, Panayiotis V Benos

**Affiliations:** 1 Department of Computational Biology, School of Medicine, University of Pittsburgh, Fifth Avenue, Pittsburgh, PA 15260, USA; 2 Center for Vaccine Research, University of Pittsburgh, Fifth Avenue, Pittsburgh, PA 15260, USA; 3 Department of Medicine, School of Medicine, University of Pittsburgh, Fifth Avenue, Pittsburgh, PA 15261, USA; 4 Department of Microbiology and Molecular Genetics, School of Medicine, University of Pittsburgh, Lothrop Street, Pittsburgh, PA 15261, USA; 5 Department of Biomedical Informatics, School of Medicine, University of Pittsburgh, Meyran Avenue, Pittsburgh, PA 15260, USA

## Abstract

**Background:**

The influenza A virus genome is composed of eight single-stranded RNA segments of negative polarity. Although the hemagglutinin and neuraminidase genes are known to play a key role in host adaptation, the polymerase genes (which encode the polymerase segments PB2, PB1, PA) and the nucleoprotein gene are also important for the efficient propagation of the virus in the host and for its adaptation to new hosts. Current efforts to understand the host-specificity of the virus have largely focused on the amino acid differences between avian and human isolates.

**Results:**

Here we show that the folding free energy of the RNA segments may play an equally important role in the evolution and host adaptation of the influenza virus. Folding free energy may affect the stability of the viral RNA and influence the rate of viral protein translation. We found that there is a clear distinction between the avian and human folding free energy distributions for the polymerase and the nucleoprotein genes, with human viruses having substantially higher folding free energy values. This difference is independent of the amino acid composition and the codon bias. Furthermore, the folding free energy values of the commonly circulating human viruses tend to shift towards higher values over the years, after they entered the human population. Finally, our results indicate that the temperature in which the cells grow affects infection efficiency.

**Conclusions:**

Our data suggest for the first time that RNA structure stability may play an important role in the emergence and host shift of influenza A virus. The fact that cell temperature affects virus propagation in mammalian cells could help identify those avian strains that pose a higher threat to humans.

## Abstract 2

RNA folding free energy is important for the evolution and host-adaptation of the influenza virus. Human virus polymerase genes are shown to have substantially higher folding free energy values than their avian counterparts.

## Background

The influenza A virus, a member of the *Orthomyxoviridae *family, is an enveloped negative single-stranded RNA virus with a genome consisting of eight individual RNA segments, each packaged into ribonucleoproteins (RNPs) [1]. RNPs are composed of four proteins, each of which is coded by a single segment. Segments 1-3 code for the three subunits of the heterotrimeric RNA-dependent RNA polymerase (PB2, PB1, and PA, respectively) and segment 5 codes for the nucleoprotein (NP), a protein that binds single-stranded RNA [2]. RNPs are sufficient for replication of the viral RNA, which leads to synthesis of positive strand complementary RNA and transcription to viral mRNA [3]. The proteins that comprise the RNPs play an important role in the adaptation of the avian viruses to humans [4], but the precise mechanism is still unclear. Recently, it was found that the three polymerase genes affect replication of avian influenza viruses [5]. Current efforts to investigate this adaptation mechanism are mainly focused on characteristic amino acid differences between avian and human genes [6]. In some cases, critical amino acid substitutions have been identified that affect species-specific virulence [7-9].

Influenza A viruses are subdivided by antigenic characterization of the hemagglutinin (HA) and neuraminidase (NA) surface glycoproteins (segments 4 and 6, respectively). HA has 16 and NA has 9 different subtypes. The most commonly circulating subtypes in the human population are A/H1N1, A/H2N2, and A/H3N2. The 1918 pandemic was caused by an A/H1N1 strain, whose polymerase genes were probably of avian origin [6]. Since then, there have been two major influenza pandemics (1957 and 1968) caused by A/H2N2 and A/H3N2 subtypes, respectively. Both strains were subject to reassortment. The human virus seems to have acquired three avian segments (HA, NA, and PB1) in the case of the 1957 pandemic, and two avian segments (HA, PB1) in the case of the 1968 pandemic [10]. The other segments are believed to have been circulating in humans since the 1918 pandemic. Currently, A/H3N2 and A/H1N1 (re-introduced into the population in 1977) are circulating in the human population [11].

Predicting the emergence of new circulating influenza strains for annual vaccine development is critical [12]. Recently, the emergence of highly pathogenic avian influenza has been of widespread concern. The majority of these outbreaks involve the direct transmission of isolates from the A/H5N1 subtype from birds to humans [13,14]. Since 2004, 385 people have been infected with H5N1 viruses, with 243 fatalities (63%). Other highly pathogenic subtypes associated with disease include A/H9N2, A/H7N7, and A/H7N3.

In this study, we investigate the role of the RNP member proteins in the propagation of the virus in birds and humans. We propose that RNA structure stability, reflected in the folding free energy, plays a critical role in overall influenza virus fitness, having an effect on replication, transmission, and spread to humans. RNA molecules with low folding energies will generally form longer stems that could potentially reduce the translation rate. Also, long stems may trigger the RNA interference mechanism, thus increasing the RNA degradation rate [15,16], which may also restrict protein production and reduce the overall number of released virions. We note, however, that long imperfect stems, especially in the 3' untranslated regions (UTRs) of the genes, can increase stability.

The discovery of differences between avian and human RNA folding energies represents a novel angle in our understanding of molecular evolutionary adaptation of influenza A virus to various hosts.

## Results

### Influenza A virus genes coding for RNP components exhibit species-specific mRNA folding energies

**Figure 1:**
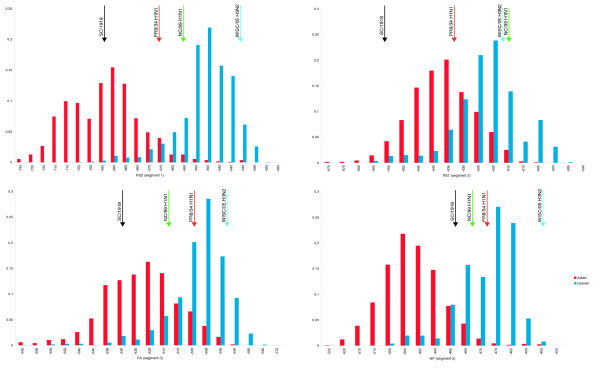
Folding free energy distributions for human and avian influenza A polymerase gene segments (in kcal/mol). The black arrows indicate the folding energies for the corresponding 1918 virus segment. Red, A/Puerto Rico/8/1934 (H1N1) (PR8/34); green, A/New Caledonia/20/1999 (H1N1) (NC/99); blue, A/Wisconsin/67/2005 (H3N2) (Wisc/05). The x-axis is the folding energy calculated by the program RNAfold [35], and the y-axis is the relative frequency of this folding energy in the viral population.

To investigate whether differences exist in the preferred folding energies of human and avian viruses, the mRNA of genes coding for PB2, PB1, PA (polymerase complex segments 1-3), and NP (segment 5) were folded as described in Materials and methods. Avian and human frequency distributions are found to be distinct in all these genes (*p *<< 0.01, Wilcoxon Rank Sum test), with segments 1 (PB2) and 5 (NP) having the most distinct distributions (Figure 1). A similar discrimination exists between the energy distributions of the avian-derived A/H5N1 strains isolated from humans and the currently circulating A/H1N1, A/H2N2 and A/H3N2 human strains (Figure S1 in Additional data file 1; *p *<< 0.01 for all segments, Wilcoxon Rank Sum test). This separation coincides with the fact that A/H1N1 and A/H3N2 strains circulate in the human population, whereas human transmission of A/H5N1 isolates is still inefficient. Avian influenza strains from other subtypes, such as A/H7N3 and A/H9N2, also exhibit folding energy preferences at the lower end of the human spectrum (data not shown).

The 1918 outbreak was the worst pandemic in recorded history. It caused severe disease with high mortality in the United States (675,000 total deaths) [10] and worldwide (50 million people) [17]. It was previously suggested that the polymerase genes of the 1918 virus were of avian origin [6]. In agreement with this hypothesis, we found that the folding energies of the polymerase genes (segments 1-3) of the 1918 strain are in the lower 1.5-4% of the human energy distributions and 6.5-67% of the avian distributions. Similarly, Kawaoka *et al. *[11] have suggested that the PB1 segment was of avian origin in the 1957 and 1968 pandemics (caused by A/H2N2 and A/H3N2 strains, respectively). We found the folding energies of the PB1 segments for all 1968 A/H3N2 isolates to be smaller than the average avian values (-655 to -635) and at the very low end of the human range, which supports the hypothesis of the avian origin of this segment. However, all the 1957 A/H2N2 isolates have folding energies in the region between the two distributions (-633 to -623), so we are not able to draw any conclusions in this case (Figure 1).

**Figure 2:**
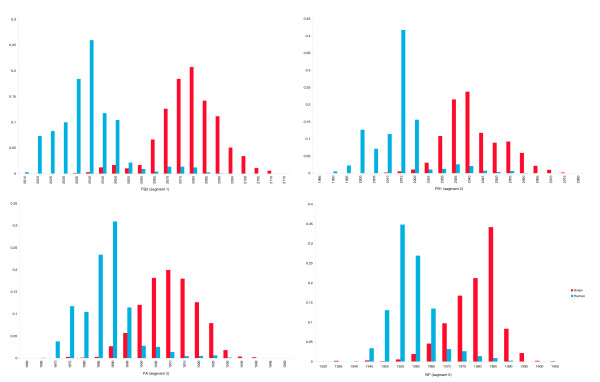
Potential hydrogen bond distribution (per segment) at all degenerate codon positions in human and avian influenza A strains. The x-axis is the number of potential hydrogen bonds per segment, while the y-axis represents the relative frequency.

Next, we examined whether the observed differences in RNA folding energy distribution between human and avian strains are a by-product of the selection performed at the protein level. Certain amino acids are known to play an important role in host-specificity. For example, Subbarao *et al. *[9] showed that a Glu to Lys substitution at position 627 of the PB2 gene is sufficient for restoring the virus's ability to replicate in Madin-Darby canine kidney (MDCK) cells. In an attempt to distinguish between the folding energy constraints and the amino acid constraints, we examined whether degenerate codon positions favored an increase or decrease in the *hydrogen bonding potential *between the viruses of the two species. Hydrogen bonding potential is defined as the number of hydrogen bonds a particular base would form if it was paired in the RNA secondary structure (see Materials and methods). While the hydrogen bond potential can not offer definite proof of whether evolution operates at the folding energy level or not, it is nevertheless indicative of the trend. If amino acid substitutions constitute the only dominant force that drives the evolution of the polymerase genes, then it would be expected that no differences would exist in the number of potential hydrogen bonds in the degenerate positions between the avian and human species. In other words, there would be no increase in the number of A or U bases in human strains compared to the avian strains at these positions. Instead, we found that degenerate positions in the avian strains contained bases with higher bonding potential than in the human strains (Figure 2). In fact, the differences between the potential hydrogen bond distributions in segments 1, 3, and 5 are similar to the distributions of the folding energies (Figure 1); and in segment 2 the differences in hydrogen bonding potential are even more profound. In all cases, the observed differences are statistically significant (*p *<< 0.01, Wilcoxon Rank Sum test). These results are in agreement with other studies that have found host-specific nucleotide bias for the influenza virus, which was attributed to host mutational bias [18,19].

Another factor that might affect the evolution of the nucleotide sequence is the codon usage bias. Each organism uses more frequently a specific set of codons for coding certain amino acid residues. In polioviruses, selection of strongly unfavorable codons can lead to reduced protein translation [20]. Could it be that this is also the case in influenza viruses and that the trend we observe in the degenerate codon positions is the result of a shift towards the host-specific codon bias? We examined this by comparing the codon frequencies of the avian and human influenza A viruses (A/H1N1, A/H3N2 and A/H5N1) to the codon frequencies of avian genes (chicken was used as representative of avian species) and human genes [21]. We found that codon frequencies are similar between the human and chicken genes (R = 0.98), and between human and avian influenza A virus genes (R > 0.97), but not between the virus genes and the animal species (R < 0.66). This suggests that the influenza polymerase genes are not under strong selection to shift towards their host codon usage preferences. In fact, this agrees with the proposed theory that, for species with small population sizes (like humans or birds), the codon usage changes are effectively neutral [22].

Based on these observations, we postulate that the folding free energy of the polymerase and NP gene segments is an important biophysical property of the segments and plays a significant role in the evolution of the virus both within the human population and in the ability of the virus to adapt to the human host when introduced from an avian source.

### Evolution of folding energies of the polymerase and NP genes

**Figure 3:**
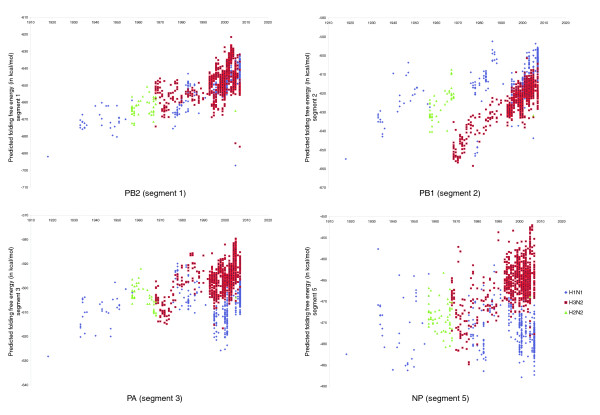
Predicted folding free energy of the human influenza A strains (polymerase genes) versus year isolated.

If there is an 'ideal range' of folding free energies for each of the polymerase and NP genes, then strains from subtypes that entered the human population at some point and circulated for many years will tend to progressively shift their folding energies towards this 'ideal' range for humans. To test this evolutionary stasis hypothesis, three of the most recently circulating human influenza A subtypes (A/H1N1, A/H3N2 and A/H2N2) were examined. We found that there was an evolutionary trend towards higher folding energies as strains from these subtypes circulated in the human population (Figure 3). Although there is no reason to expect that the changes in the folding energy will correlate linearly with the year, we observe in fact such correlation for parts of the evolutionary trend. For example, segment 1 (PB2) of the A/H1N1 strains isolated since 1918 shows a shift towards higher folding energies, which continues after the strain's re-emergence in 1977 (R = 0.80, *p *<< 0.01). Segment 2 (PB1) also shows some linear correlation for the years 1918-1956 (R = 0.77, *p *= 10^-6^), when the strain was replaced by A/H2N2. During the years that the A/H2N2 strain was in circulation (1957-1967), we observe a weak linear correlation of the folding energies with the year (R = 0.69, *p *= 10^-6^). In 1968 the A/H2N2 strain was replaced by an A/H3N2 strain. The newly introduced segment 2 (from bird viruses) continued having strong correlation of the folding energies with the year until 1998 (R = 0.89, *p *<< 0.01). Finally, for segment 3 (PA) of the A/H3N2 strain, we observe linear correlation in the years 1968-1985 (R = 0.75, *p *<< 0.01). Notably, none of the avian strains shows such a pattern over the same time period (Figure S4 in Additional data file 1).

### RNA folding energy and cell temperature

One of the factors that determine RNA folding energy is temperature. If viral RNA and mRNA folding energy affects the efficiency of viral infection and replication, then one would expect that virulence will vary according to the temperature that cells are incubated at and the folding energy of the viral segments. To further investigate this hypothesis, MDCK cells were slowly adapted for growth at two temperatures higher than 37°C (39°C and 40°C) as described in Materials and methods. The slow adaptation allowed cells to adjust to higher temperatures, thus minimizing the risk of injury due to heat shock. The adapted cells showed no difference in their growth rate. Further support for the regular growth of the cells comes from the fact that one of the mammalian influenza viruses, A/Puerto Rico/8/1934 (H1N1) (PR8/34), was able to replicate equally well in MDCK cells incubated at all temperatures in the 37-40°C range (Table 1).

**Table 1:** Viral titer (PFU/ml) for A/Puerto Rico/8/1934 (PR8/34) and A/New Caledonia/20/1999 (NC/99) H1N1 strains, and for A/Wisconsin/67/2005 (Wisc/05) H3N2 strain

	PR8/34 A/H1N1		NC/99 A/H1N1		Wisc/05 A/H3N2	
			
	48 h	96 h	48 h	96 h	48 h	72 h
37°C	2.5 × 10^8^	4.2 × 10^9^	1.0 × 10^5^	1.1 × 10^9^	1.0 × 10^5^	>10^6^
39°C	1.7 × 10^8^	7.4 × 10^9^	<100	<10^4^	3.0 × 10^3^	3.2 × 10^6^
40°C	1.0 × 10^8^	2.0 × 10^8^	<100	<10^4^	<100	<100

The folding energies for segments 1-3, and 5 are: PR8/34, [-671.33, -633.85, -604.73, -473.22]; NC/99, [-658.78, -615.39, -611.74, -477.67]; Wisc/05, [-637.74, -617.08, -593.41, -455.20].

MDCK cells, incubated at 37°C, 39°C and 40°C, were infected with one of two A/H1N1 human strains - A/New Caledonia/20/1999 (H1N1) (NC/99), and A/Puerto Rico/8/1934 (H1N1) (PR8/34) - or one A/H3N2 human strain - A/Wisconsin/67/2005 (H3N2) (Wisc/05). Viral replication was measured by plaque assay at various time points post-infection. What becomes apparent from the results in Table 1 is that the viral titer generally decreases with increased temperature, and the rate of decrease depends on the virus. Both NC/99 and Wisc/05 produced no viral plaques at 40°C, but Wisc/05 produced plaques at 39°C, whereas NC/99 did not. Finally, PR8/34 was found to replicate efficiently at all three temperatures. Notably, all four PR8/34 segments (segments 1-3, and 5) have folding energy values in the range between the human and avian average values (Figure 1). Compared to PR8/34, NC/99 has higher folding energies for segments 1 and 2 and similar or slightly lower energies for segments 3 and 5. However, the folding energies of segments 1 and 2 of NC/99 are at the extreme end of the avian distribution, which might explain its inability to replicate efficiently at higher temperatures, as indicated by the viral titer values (Table 1). All four segments of Wisc/05 have RNA folding free energy values higher than the average for human influenza A viruses (Figure 1). So, based on the hypothesis that cell temperature affects viral replication through the folding energy of the polymerase genes, Wisc/05 is expected to replicate more efficiently at 37°C than at higher temperatures. Consistent with that hypothesis, no plaques were observed when MDCK cells, infected with Wisc/05, were incubated at 40°C, and there were fewer plaques on MDCK cells incubated at 39°C compared to MDCK cells incubated at 37°C (Table 1).

### Ability of the H5N1 influenza A virus to become established in the human population

**Figure 4:**
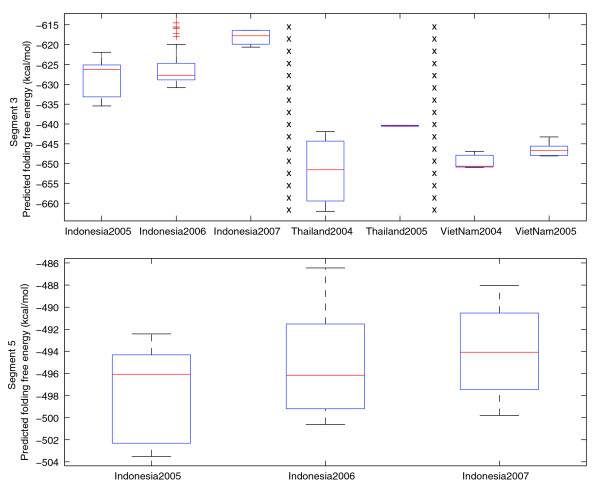
Predicted folding free energy of human A/H5N1 cases (polymerase gene segments 3 and 5) arranged by location and year of outbreak.

The ability of an avian virus to jump from the bird population directly to the human population has been recorded for the A/H5N1, A/H7N3, A/H7N2, and A/H9N2 subtypes [23,24]. Most of these human outbreaks have been limited to a single round of infection from birds to humans with little or no human-to-human transmission. Nevertheless, the A/H5N1 human outbreaks have occurred in at least 16 countries across 3 continents since 1997 [25], and strains of the avian A/H5N1 subtype are considered to be a threat to humans because of their pandemic potential [26]. For this reason, we decided to further examine the folding energies for avian A/H5N1 isolates. Box plots of the folding energies of segments 3 and 5 were calculated for all observations from the same region when data existed for two or more consecutive years (Figure 4). Differences in the yearly plots are not statistically significant for all but one of them (Indonesia population, segment 5, *p *= 0.04). This is expected for changes occurring over short periods of time. Nevertheless, these plots show a clear trend towards higher energies from year to year, which would favor adaptation to human hosts according to our hypothesis. For segments 1 and 2 no such trend was observed, but we note that the vast majority of segment 1 and 2 sequences from these regions have folding energies already in the human spectrum (data not shown).

We also analyzed the folding energies for five A/H5N1 strains that are currently recommended by the World Health Organization for the production of vaccines against potential pandemic A/H5N1 influenza. The 1918 virus was used in this analysis as a low energy limit for the virus to be able to efficiently propagate in humans. The folding energy values of the 1918 virus are among the smallest observed in human viruses, and the virus caused one of the worst pandemics. In all but one case, segments 1-3 of the A/H5N1 viruses had higher folding energies than the corresponding segments of the 1918 strain (Table S1 in Additional data file 1). The exception is segment 3 of the A/Vietnam/1203/2004 (VN/04) H5N1 strain, with a predicted folding free energy of -651 kcal/mol compared to the 1918 value of -628 kcal/mol. These data suggest that, as far as segments 1-3 are concerned, all but one A/H5N1 strain analyzed (VN/04) have the potential to contribute to efficient transmission from human-to-human and, hence, the establishment of the virus in the human population.

Hatta *et al. *[7] studied the virulence of two H5N1 influenza A strains with respect to residue 627 of the PB2 protein. They found that strain A/Vietnam/1203/2004 with Lys at position 627 of PB2 was three times more efficient in infecting mice cells than A/Vietnam/1204/2004, which has Glu at this position (MLD_50 _of 0.7 compared to 2.1). We folded the two PB2 segments and found them to differ by approximately 2 kcal/mol, with A/Vietnam/1203/2004 having higher energy (-682 versus -684). Although the difference is small, we note that both strains have PB2 folding energies at the extreme low end of the human distribution (Figure 1). It is possible that at distribution extremes, even small differences can give the virus an evolutionary advantage. In addition, Hatta *et al. *[7] performed site-directed mutagenesis and replaced the amino acid at position PB2-627 in each of the strains with the amino acid of the other strain. The new strains, VN1203PB2-627E and VN1204PB2-627K, had measured MLD_50 _values of 67.6 and 0.6, respectively. Interestingly, the corresponding folding energies of these mutants are -684.2 (VN1203PB2-627E) and -681.7 (VN1204PB2-627K). It is easy to see that for all four proteins (initial isolates and mutants), the order of the MLD_50 _values coincides with the order of the negative folding energy values (rank correlation coefficient R = -1). In fact, if we exclude mutant VN1203PB2-627E from the analysis (because, practically, it does not infect the cells), the remaining three segments exhibit a strong anti-correlation between MLD_50 _and folding energy values (R = -0.97). In other words, in this case, the virulence of the virus with respect to PB2 seems to be associated with how close its folding energy is to the human average (Figure 1), with the segments closer to the average being more virulent.

## Discussion

In this study, we have analyzed a biophysical property of the RNA segments of the influenza A virus: the folding free energy. We show that folding free energies of the RNP complex genes (PB2, PB1, PA and NP) differ between avian and human viruses and between seasonal human viruses and A/H5N1 viruses isolated from humans. The fact that the other segments do not show such drastic folding energy preferences (data not shown) may reflect the importance of the polymerase genes in escaping the host's cellular response [27].

The choice of focusing on the coding regions (or open reading frames (ORFs)) rather than on the complete segments was dictated by the fact that a large percentage of the sequences in the database (20-48%, depending on the segment and the host species) lack information about the 5' UTR, the 3' UTR, or both. Thus, analyzing the coding regions provided the largest common dataset. Given the small length of the non-coding regions (compared to the ORFs), their effect on the analysis of the folding energies is expected to be small. In other words, it is reasonable to believe that the trends observed in the analysis of the coding regions are representative of the phenomenon seen for the whole segments. However, non-coding regions can be important for viral RNA replication [28], hence affecting virulence. For example, certain 5' UTRs may enhance the translation efficiency or some 3' UTRs may contain targets for microRNA genes from the host. But these phenomena are independent of the folding energies, so their contribution to virulence is similar to the contribution of HA, NA or the other non-RNP genes, and hence not a subject of our analysis.

Based on the folding energy distributions of the human and avian strains, we postulated that the avian virus segments may fold into a more 'rigid' structure in human cells than in avian cells. Such structure is expected to have long stems. Long stems with no mismatches can result in slower translation rates or increased degradation rates of the mRNA molecules [15,16]. Either case can result in a reduction in viral fitness. We showed that, in the case of MDCK cells, human strains NC/99 (A/H1N1) and Wisc/05 (A/H3N2), with folding energies of the polymerase genes and NP segment largely in the human range, propagated efficiently at 37°C, but their propagation was diminished at higher temperatures. In contrast, strain PR8/34 (A/H1N1), with folding energies in the region between human and avian average values, propagated equally well at all temperatures. This shows that the cells that were slowly adapted in higher temperatures have no difficulty in propagating human influenza A viruses. It also shows that viruses with high folding energies (in the human range) may have difficulties propagating in birds. Whether avian viruses with very low energies have difficulties propagating in human cells remains to be seen. We note, however, that if this is true, then the host's body temperature may impose an additional barrier to cross-species transmission. Finally, we found that the RNA folding free energy of the A/Vietnam/1203/2004 and A/Vietnam/1204/2004 H5N1 viruses and the mutant VN1204PB2-627K show a nearly perfect inverse correlation with the measured MLD_50 _values (R = -0.97). The effect of the folding energy on the evolution of the virus appears to be independent of the concurrent amino acid changes in the polymerase and NP genes, and independent of the codon usage bias. In addition, human influenza A strains have increasingly higher folding energies over time (within a certain range), especially when their folding energy starting points are close to the avian range.

Taken together, these results suggest that the folding free energy of the RNA molecules of the polymerase segments is an important factor in the evolution of the influenza A virus. Previous research in this area was focused on amino acid changes, especially in the HA, NA, and PB2 genes [7-9], where a number of mutations were found to be critical for host adaptation of the virus. The fact that the 1918 A/H1N1 has segments 1-3 with RNA folding free energies in the lowest part of the human spectrum (Figure 1) is indicative of the importance of the NA and HA genes in the success of replication and host adaptation [29].

In agreement with previous studies [6], our data support the idea that the polymerase genes (PB2, PB1, PA) of the 1918 A/H1N1 virus were of avian origin, since they are outside of the spectrum of the A/H1N1 folding energies and in the lower spectrum of folding energies of all human viruses. Also, our results support the hypothesis that the PB1 segment in the 1968 pandemic (but not necessarily in the 1957 pandemic) was of avian origin. The possibility of an avian influenza A virus strain crossing the host barrier and successfully propagating in humans has been controversial [26,30]. So far, cases of avian-to-human transmission are limited, both in number and virulence. From the folding free energy perspective and in light of the results above, we can postulate that avian viruses whose RNP complex genes have folding energies in the corresponding human spectra will have increased chances to establish themselves in the human population. So far, no avian virus has been found with all its RNP segments in the human range, although this might reflect gaps in the sequence data. Nevertheless, should a re-assortment and the necessary amino acid changes occur in HA segments coding for glycoproteins with specificity for human receptors (sialic acid alpha-2,6-galactose), it is possible that an avian A/H5N1 strain may cause a pandemic in humans.

To our knowledge, this is the first time that RNA folding was identified as a factor in the evolution and adaptation of the influenza A virus. Taken together, our results are consistent with the hypothesis that the host's body temperature may play an important role in the host adaptation of a virus, although clearly more experimentation is required. Interestingly, the folding free energy distribution of the swine viruses is intermediate between the avian and human distributions (Figure S3 in Additional data file 1) and the swine is known as an intermediate host (possibly as a 'mixing vessel') for avian viruses jumping into humans. The swine's mean body temperature range is 37.8-38.6°C [31], which is also intermediate between avian and human body temperature ranges. Also, the folding free energy distributions of the avian viral genes become indistinguishable from the human distributions if the avian genes are folded at 38°C (Figure S2 in Additional data file 1). Having said that, the evolution of the influenza A virus is complicated and the folding free energy hypothesis can not explain all observations. The RNP complex genes of the 1918 virus, for example, have very small folding free energies compared to the rest of the human viral genes and still caused one of the most devastating pandemics in history. Waterfowl birds present another interesting case. Influenza viruses isolated from chickens can seamlessly circulate in waterfowl birds, although the latter generally have higher average body temperatures [32]. On the other hand, the body temperature of waterfowl birds varies substantially between different organs, as well as the bird's activity during the day [33], which adds to the complexity of the evolutionary forces shaping the propagation of the virus.

## Conclusions

This study is mainly based on computational analysis of the available influenza data. The results support the intriguing hypothesis that the RNA folding free energy of the polymerase genes plays an important role in the evolution and host specificity of the influenza A virus. We hope these results will stimulate further biochemical research on the subject. For example, isogenic chimeric viruses with different polymerase genes, but the same HA and NA segments, can be used to further test the hypothesis of viral replication dependence on temperature in human and avian cells. One of the challenges will be to combine amino acid composition, mRNA folding energy and other factors in a single evolutionary analysis framework. To that extent, work on animal models is necessary to help understand the mechanism by which RNA folding free energies shape the adaptation of the influenza virus from birds to humans.

## Materials and methods

### Sequences and codon usage tables

Influenza A sequences, isolated from human, and avian species, were downloaded from NCBI's Influenza Virus Resource Database [34] in March 2008. For the calculation of the folding energy distributions, we used all available human and avian strains with at least one complete ORF sequence (human: A/H1N1, A/H1N2, A/H2N2, A/H3N2, A/H5N1, A/H7N3, A/H9N2; avian: A/H1N1, A/H1N2, A/H1N3, A/H1N5, A/H1N6, A/H1N9, A/H2N1, A/H2N2, A/H2N3, A/H2N4, A/H2N5, A/H2N7, A/H2N8, A/H2N9, A/H3N1, A/H3N2, A/H3N3, A/H3N4, A/H3N5, A/H3N6, A/H3N8, A/H4N1, A/H4N2, A/H4N3, A/H4N4, A/H4N5, A/H4N6, A/H4N8, A/H4N9, A/H5N1, A/H5N2, A/H5N3, A/H5N6, A/H5N7, A/H5N8, A/H5N9, A/H6N1, A/H6N2, A/H6N3, A/H6N4, A/H6N5, A/H6N6, A/H6N8, A/H6N9, A/H7N1, A/H7N2, A/H7N3, A/H7N4, A/H7N5, A/H7N7, A/H7N8, A/H7N9, A/H8N2, A/H8N4, A/H9N1, A/H9N2, A/H9N4, A/H9N5, A/H9N6, A/H10N1, A/H10N2, A/H10N3, A/H10N4, A/H10N5, A/H10N6, A/H10N7, A/H10N8, A/H10N9, A/H11N1, A/H11N2, A/H11N3, A/H11N6, A/H11N8, A/H11N9, A/H12N1, A/H12N4, A/H12N5, A/H12N9, A/H13N2, A/H13N3, A/H13N6, A/H13N9, A/H14N5, A/H14N6, A/H15N2, A/H15N8, A/H15N9, A/H16N3). The vast majority of the bird strains were isolated from chicken and duck (about equal number of sequences from each species). For the analysis of the folding free energies versus time, we used the more commonly circulating human strains (A/H1N1, A/H2N2, and A/H3N2). Only sequences corresponding to the complete ORF of each segment were considered for reasons we describe in the text. A complete ORF was defined as having both a start and a stop codon. The position of the start codon was determined by a multiple protein sequence alignment of each segment in each species, for a total of eight multiple alignments (four genes, two species). There are no length differences between the corresponding human and avian segments, although the four segments vary between them in terms of protein length (340-759 amino acids) and GC content (42.7-47% for human and 43-47.5% for avian mRNAs). If two or more segment sequences were identical at the nucleotide level, only one of them was used in the analysis. As we explained above, the choice of focusing on the ORF was dictated by the fact that the majority of the sequences in the database contain partial or no non-coding sequence. Thus, analyzing only the ORFs provided the largest possible dataset. Codon usage tables for human and chicken were obtained from the current version (September 2007) of the Codon Usage Tabulated from the GenBank (CUTG) database [21].

### RNA folding

The folding free energy of each segment was computed using the Vienna RNA (version 1.6.5) package's RNAfold program [35], with the default parameters, save temperature, which was varied as we describe in the text.

### Hydrogen bonding potential

The hydrogen bonding potential on the degenerate codon positions was calculated by assigning two hydrogen bonds to an A or U, and three to a C or G in every degenerate codon position. G•U pairs were not considered in this analysis, since it would have made it difficult to assign a number of hydrogen bonds to Gs and Us if the structure was unknown (or differed depending on the molecule). The bond assignment is based on the primary sequence, not the predicted secondary structure.

### MDCK cell adaptation and plaque assays

MDCK cells were adapted for efficient growth at temperatures higher than 37°C (namely, 39°C, and 40°C). To minimize cell injury due to heat-shock and to ensure that cells are responsive to the viruses, we passaged them at higher temperatures gradually over a period of 21 days. MDCK cells were propagated in Dulbecco's modified Eagle's medium (DMEM) supplemented with 10% fetal calf serum in 5% CO_2 _and the temperature was increased by 0.2°C every three days. Aliquots of cells adapted for efficient growth at 39°C and 40°C were frozen at -80°C. Viruses were propagated and harvested from supernatants in cells grown at 37°C. MDCK cells plated in 6-well tissue culture plates were inoculated with 0.1 ml of virus serially diluted in DMEM. Virus was adsorbed to cells for 1 h, with shaking every 15 minutes. Wells were overlaid with 1.6% w/v Bacto agar (DIFCO, BD Diagnostic Systems, Palo Alto, CA, USA) mixed 1:1 with L-15 media (Cambrex, East Rutherford, NJ, USA) containing antibiotics and fungizone, with 0.6 μg/ml trypsin (Sigma, St Louis, MO, USA). Plates were inverted and incubated for 2-3 days. Wells were then overlaid with 1.8% w/v Bacto agar mixed 1:1 with 2× Medium 199 containing 0.05 mg/ml neutral red, and plates were incubated for two additional days to visualize plaques. Plaques were counted and compared to uninfected cells. The ability of the PR8/34 (A/H1N1) virus to infect cells equally efficiently at all temperatures further suggests that any potential heat-shock effect is negligible.

## Abbreviations

DMEM: Dulbecco's modified Eagle's medium; HA: hemagglutinin; MDCK: Madin-Darby canine kidney cells; NA: neuraminidase; NP: nucleoprotein; ORF: open reading frame; RNP: ribonucleoprotein; UTR: untranslated region.

## Authors' contributions

PVB and RB-S conceived and designed the study, performed the computational analyses, and analyzed the data. DMC and CJC infected cells and collected viral titer data under the direction of TMR. PVB, RB-S, TMR and EG wrote the paper.

## Additional data files

The following additional data are available with the online version of this paper. Additional data file 1 contains four figures showing various plots of folding energies (referenced in the main text) and one table listing the folding energies of vaccine strains WHO and CDC use against H5 influenza.

## Supplementary Material

Additional data file 1 Plots of folding energies of vaccine strains WHO and CDC use against H5 influenza Click here for file
