# Supplementary material for: The role of RNA folding free energy in the evolution of the polymerase genes of the influenza A virus
Source: Genome Biol. 2009 Feb 12;10(2):R18. doi: 10.1186/gb-2009-10-2-r18 (PMC2688270; doi:10.1186/gb-2009-10-2-r18)

SUPPLEMENTARY MATERIALS for the paper

Rachel Brower-Sinning, Donald M. Carter, Corey J. Crevar, Elodie Ghedin, Ted M. Ross, Panayiotis V. Benos, "***The role of RNA folding free energy in the evolution of the polymerase genes of the influenza A virus***"

SUPPLEMENTARY TABLE

Table S1. Predicted folding energies of the five A/H5N1 strains that WHO and CDC use as vaccine strains against H5 influenza. For comparison purposes, the values for the 1918 strain were included. **Bold letters** indicate the smallest value of the segment; **red letters** indicate a smaller folding energy than the corresponding 1918 segment.

	Segment			
	1	2	3	5
A/Viet Nam/1203/2004 (VN/04)	-682.38	-627.92	**-650.94**	-495.72
A/Indonesia/05/2005 (Indo/05)	-680.61	-618.52	-625.42	-494.32
A/Hong Kong/156/1997	-652.82	-640.43	-612.30	-487.12
A/Hong Kong/483/1997	-660.72	-642.36	-622.63	-489.30
A/Hong Kong/486/1997	-657.71	-637.76	-609.70	**-497.60**
**A/South Carolina/1/1918**	**-691.92**	**-654.75**	-628.20	-482.42

LEGENDS for SUPPLEMENTARY FIGURES

Figure S1. Folding free energies for all human influenza A polymerase gene segments (in kcal/mol). The arrows indicate the folding energies for the corresponding A/South Carolina/1/1918 (H1N1) virus segments.

Figure S2. Distribution of folding free energy (in kcal/mol) for human, and avian influenza A strains folded at the body temperature of the host.

Figure S3. Folding free energy distributions for human, swine and avian influenza A polymerase gene segments (in kcal/mol).

Figure S4. Predicted folding free energy of the avian influenza A strains (polymerase genes) *vs.* year isolated.

Supplementary Figure S1: Folding free energies for all human influenza A polymerase gene segments (in kcal/mol). The arrows indicate the folding energies for the corresponding 1918 virus segments.

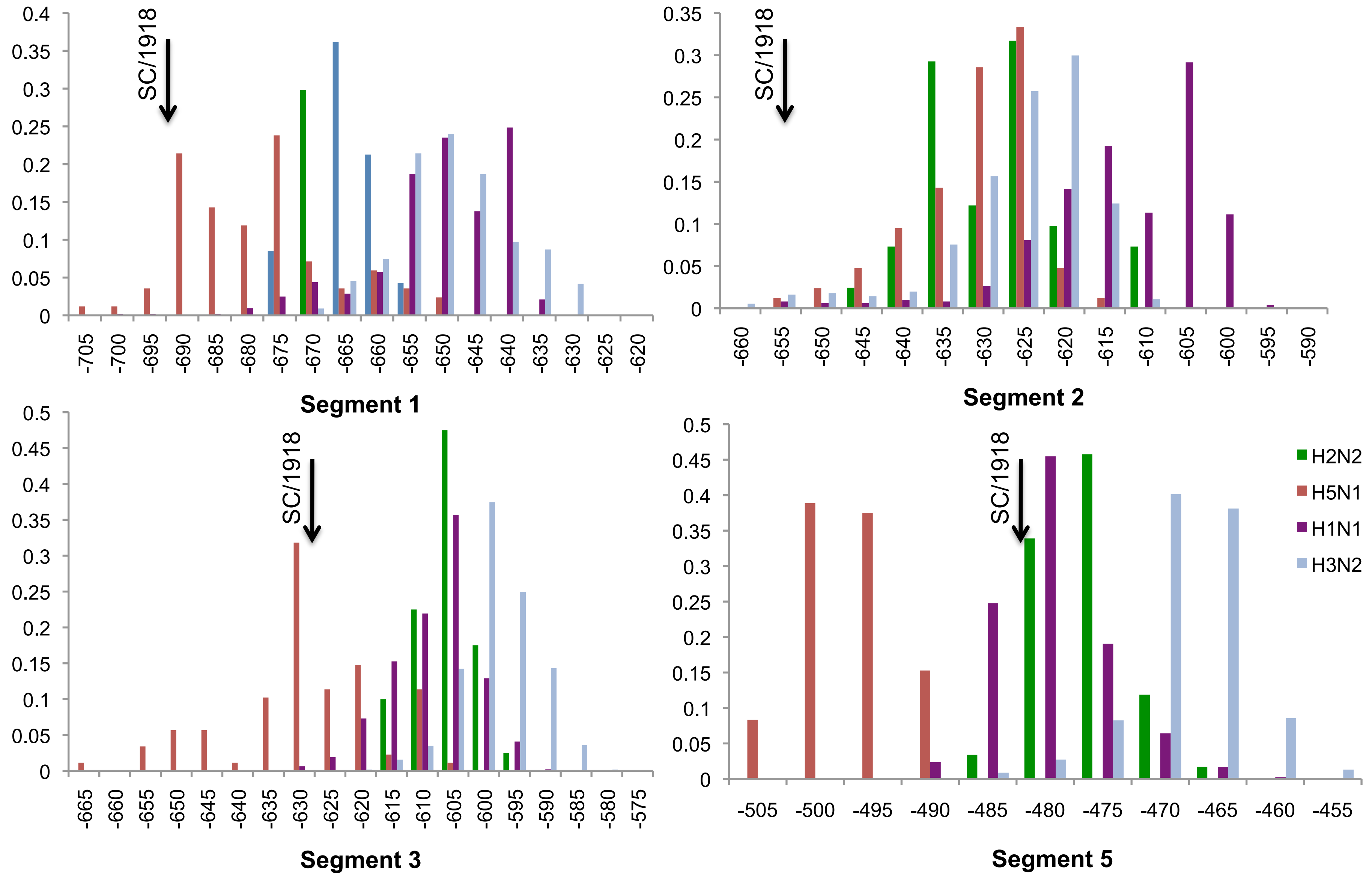

Supplementary Figure S2: Distribution of folding free energy (in kcal/mol) for human, and avian influenza A strains folded at the body temperature of the host.

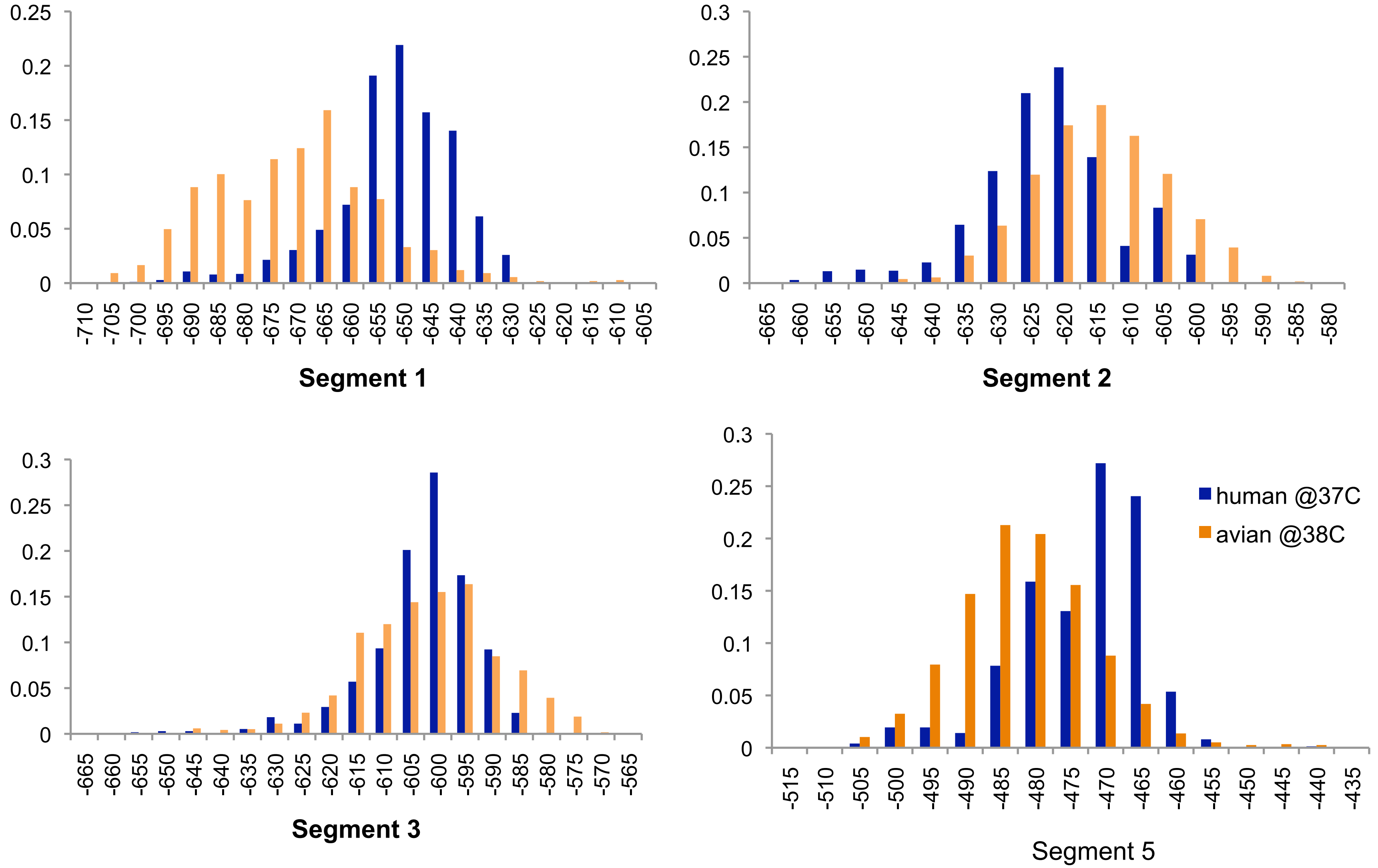

Supplementary Figure S3: Folding free energy distributions for human, swine and avian influenza A polymerase gene segments (in kcal/mol).

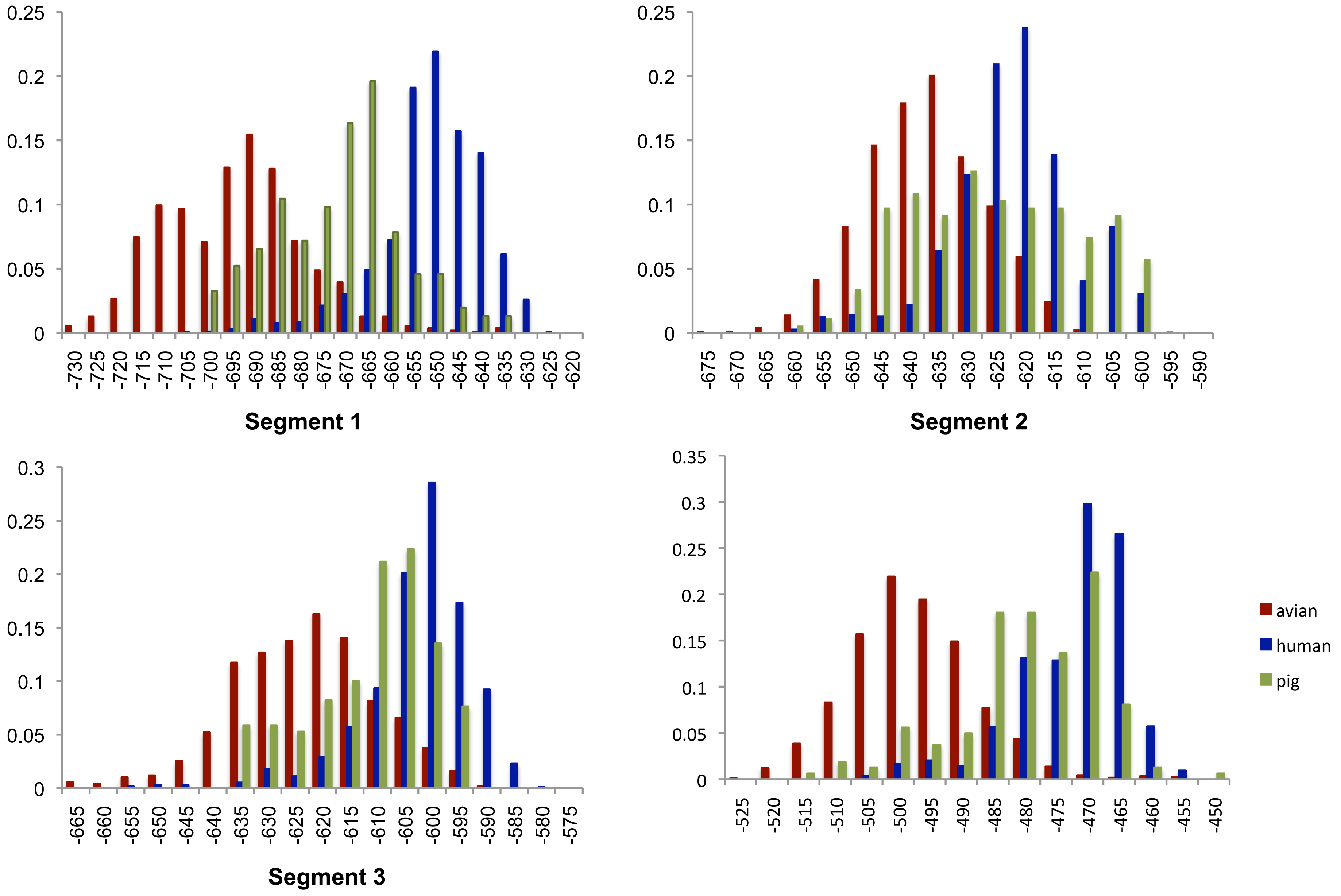

Supplementary Figure S4: Predicted folding free energy of the avian influenza A strains (polymerase genes) *vs.* year isolated.

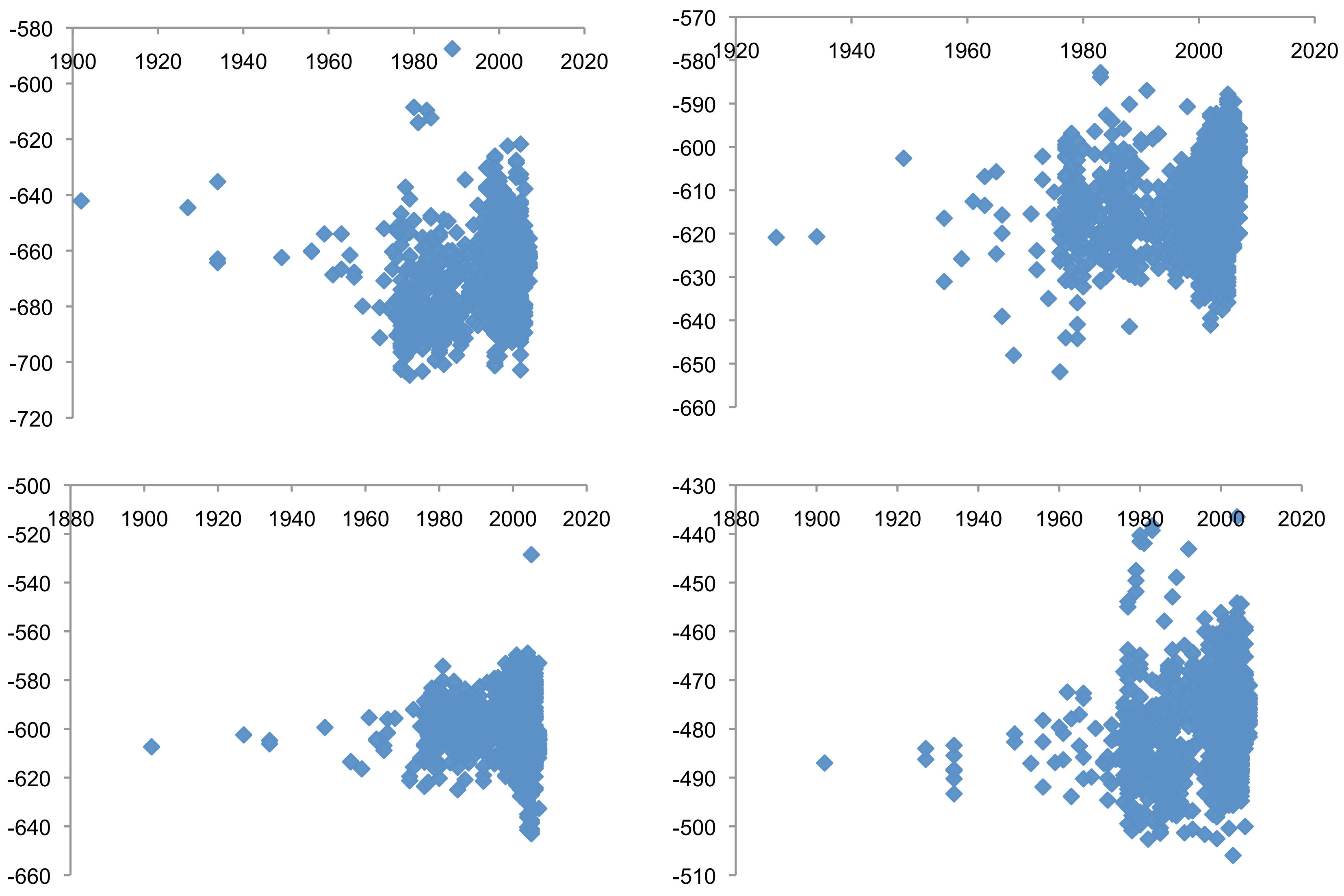

Supplement: Additional data file 1 — Plots of folding energies of vaccine strains WHO and CDC use against H5 influenza [file gb-2009-10-2-r18-S1.pdf]
